# Relationship Between Body Composition With Carotid Intima Media Thickness in Young Adults: Tehran Lipid and Glucose Study

**DOI:** 10.1002/edm2.70173

**Published:** 2026-02-28

**Authors:** Mohammad Nikoohemmat, Behnaz Abiri, Majid Valizadeh, Maryam Mahdavi, Pooneh Dehghan, Fereidoun Azizi, Farhad Hosseinpanah

**Affiliations:** ^1^ Obesity Research Center, Research Institute for Metabolic and Obesity Disorders, Research Institute for Endocrine Sciences Shahid Beheshti University of Medical Sciences Tehran Iran; ^2^ Department of Imaging, Taleghani Hospital Shahid Beheshti University of Medical Sciences Tehran Iran; ^3^ Endocrine Research Center, Research Institute for Endocrine Sciences Shahid Beheshti University of Medical Science Tehran Iran

**Keywords:** body composition, carotid intima media thickness, cIMT, young adults

## Abstract

**Background:**

Body composition, particularly the interplay between skeletal muscle mass and adiposity, has gained attention for its potential impact on cardiovascular health. This study aimed to examine the relationship between body composition and carotid artery intima‐media thickness (cIMT) as an indicator of early atherosclerosis.

**Methods:**

This cross‐sectional study was conducted in the Tehran Lipid and Glucose Study (TLGS) cohort, focusing on young adults. Anthropometric measurements, laboratory tests and cIMT assessments were performed. Body composition was assessed using bioelectrical impedance analysis (BIA). The association between skeletal muscle mass index (SMMI) and skeletal muscle mass (SMM) with cIMT was analysed using linear and logistic regression models, adjusted for relevant covariates.

**Results:**

A total of 795 participants (361 women, 434 men) were included in the study. Higher SMMI and SMM quartiles were associated with increased cIMT and a higher risk of high cIMT (above the 75th percentile). For instance, in the age‐adjusted model, the highest quartile (Q4) of SMMI was associated with high cIMT with an OR of 1.80 (95% CI, 1.02–3.20) in men and an OR of 2.17 (95% CI, 1.14–4.13) in women. While these associations were significant after adjustment for age, further adjustment for cardiovascular risk factors attenuated some associations, particularly for SMMI quartiles. However, SMMI and SMM as continuous variables remained positively and significantly associated with cIMT in both sexes (e.g., for fully‐adjusted continuous SMMI: *β* = 0.010 in men, *β* = 0.013 in women), indicating a persistent linear relationship independent of major confounders.

**Conclusion:**

In young adults, higher skeletal muscle mass was independently associated with greater cIMT, suggesting it may signal early vascular changes rather than protection, especially when coinciding with higher adiposity. This underscores the need to assess overall body composition and for future longitudinal studies to determine causality.

## Graphical abstract

Our study of 795 young adults found that higher skeletal muscle mass was linked to thicker carotid arteries (increased cIMT), even after considering body fat and other risks. The takeaway: in early adulthood, higher muscle mass—often paired with higher body fat—may signal early vascular changes rather than protection. Cardiovascular health strategies should target optimal overall body composition, not just increasing muscle.

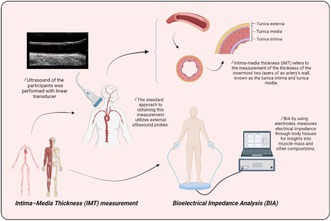

## 1 Introduction

Body composition, specifically the interplay between skeletal muscle mass and adiposity, has garnered significant attention in recent years owing to its potential impact on cardiovascular health [1, 2]. However, the exact mechanisms that lead to metabolic and vascular complications remain unclear [3]. Previous epidemiological studies of the combined effects of lean muscle mass and obesity on metabolic disorders and heart disease have yielded conflicting results [4, 5, 6]. Therefore, investigating the indicators related to the development of this disorder can be very important in detecting the occurrence of these factors and preventing their influence on the progression of atherosclerotic disorders [7].

Recent research has shown that low skeletal muscle mass is associated with several health problems, including type 2 diabetes, metabolic syndrome, non‐alcoholic fatty liver disease, and an increased risk of cardiovascular disease and mortality [8, 9, 10, 11]. Of course, it should be noted that these findings have been observed for the general population, including young adults, in addition to the elderly [8, 10, 12]. However, the exact mechanism remains to be fully understood. Therefore, further studies should be conducted on the relationship between skeletal muscle mass and carotid intima‐media thickness (cIMT). However, maintaining skeletal muscle mass is necessary for proper physical movement and various metabolic and homeostatic functions. Some studies have reported conflicting results regarding the potential association between sarcopenia markers and preclinical vascular disease [13, 14, 15, 16].

Several cohort and prospective studies have demonstrated a strong link between increased cIMT and the likelihood of developing future cardiovascular diseases, such as stroke, myocardial infarction (MI) and cardiac mortality [17, 18, 19]. The B‐mode ultrasound measurement of cIMT has proven to be a valuable screening tool for the early detection of atherosclerosis in asymptomatic individuals [20, 21].

According to the recent recommendation of the Asian Working Group for Sarcopenia (AWGS) 2019 to studies using SMM compared to BMI (BMI adjusted), few previous studies have investigated the association of SMM and SMMI with cIMT in young adults [22, 23, 24]. However, the relationship between muscle mass and CVD is not yet fully understood. Therefore, considering that the relationship between muscle mass and CVD is yet to be entirely determined, this cross‐sectional study aimed to investigate the association between body composition—specifically skeletal muscle mass (SMM) and skeletal muscle mass index (SMMI)—and cIMT, a marker of subclinical atherosclerosis, in a population‐based sample of young adults from the Tehran Lipid and Glucose Study (TLGS). A secondary aim was to explore whether any observed association was independent of overall adiposity, assessed by percent body fat.

## 2 Materials and Methods

### 2.1 Study Population and Inclusion Criteria

This cross‐sectional study, conducted in the frame of Tehran Lipid and Glucose Study (TLGS) cohort study framework. TLGS is a prospective, community‐based study currently underway to determine risk factors and outcomes of non‐communicable diseases [25]. To conduct the study, the researchers utilised a multi‐stage random sampling method to select participants over three years old. The selection process was carried out in Tehran's 13th district. They were followed up every three years to update anthropometric examinations, demographic information, biochemical status, clinical characteristics, and lifestyle data. In the first phase, a baseline cross‐sectional survey was conducted from 1999 to 2001; a prospective follow‐up survey was performed from 2002 to 2005 (the second phase). In the first stage, a baseline cross‐sectional survey was conducted from 1999 to 2001. In the following order, a prospective follow‐up study was conducted from 2002 to 2005 (Phase II). Then phases 3 to 5 were run every three years until 2016, and finally, 2016 to 2019 (Phase VI) and 2019 to 2021 (Phase VII) were implemented as the next stages of the study.

Of all the individuals who participated in the initial stage of the study and attended all subsequent follow‐ups up to the seventh stage, 2641 individuals were eligible for the cIMT assessment. Out of this number, 1455 participants were present for cIMT measurement, of which 806 aged between 20 to 40 years met the inclusion criteria for our study. All analyses were performed separately for men and women to account for fundamental differences in body composition and cardiovascular risk profiles.

The measurement of cIMT took place between February 2017 and October 2019. The exclusion criteria of this study included people with cancer (*n* = 3), chronic use of diuretics (*n* = 10), chronic use of corticosteroids (*n* = 33), and those who were pregnant (*n* = 13). Additionally, the study excluded 585 participants who did not have BIA data measured in either phase 6 or 7. The Institutional Ethics Committee of the Endocrinology Research Institute, affiliated with Shahid Beheshti University of Medical Sciences, approved the study's design (code: IR.SBMU.ENDOCRINE.REC.1401.079). All the Declaration of Helsinki tenets were followed, and the participants signed written informed consent forms.

### 2.2 Anthropometric Assessment, Laboratory Measurement, Assessment of the cIMT of Carotid Artery and BIA

#### 2.2.1 Anthropometric Measurements

Qualified healthcare professionals performed anthropometric measurements according to standard protocols [25]. The participant's weight was measured using a digital scale while minimally clothed and without shoes. The scale provided measurements to the nearest 0.1 kg (Seca707; range 0.1–150 kg, Hanover, MD, USA). A meter tape stadiometer was used to measure the participant's height while they stood next to a wall with their shoulders in a natural alignment. Their waist circumference (WC) was measured at the narrowest point between the iliac crest and the lowest rib using a non‐stretch tape while standing without any pressure on the body surface and exhaling at the end. Also, the cut‐off for abdominal obesity was considered waist circumference ≥ 90 cm. Height and waist circumference were recorded with an accuracy of 0.1 cm. BMI was calculated by dividing weight (in kilograms) by the square of height (in square meters), which was, according to international cut‐off points, the basis for dividing people into normal weight (BMI < 25), overweight (25 ≤ BMI < 30), and obese (BMI ≥ 30) groups.

A doctor measured the systolic and diastolic blood pressure using a standard mercury sphygmomanometer calibrated by the Iranian Standard and Industrial Research Institute. This measurement was taken after the participant had been sitting for 15 min. Then, the average of 2 blood pressure measurements with an interval of 30 s was reported as the participant's blood pressure. It should be noted that the pressure of the right brachial artery, which was at the level of the heart, was measured.

#### 2.2.2 Laboratory Measurements

Per the standard procedure, the participants' blood samples were collected between 7 am and 9 am, following a fasting period of 12 to 14 h. Samples were centrifuged for 30 to 45 min after collection. The TLGS research laboratory evaluated the serum and centrifuged blood samples collected on the same day to measure and analyse the concentrations of lipids and fasting plasma glucose (FPG). The laboratory utilised kits that are accessible in the Iranian market (provided by Pars Azmoun Company in Tehran, Iran) and were adjusted to be compatible with a Selectra 2 automatic analyser.

The enzyme colorimetric method with glucose oxidase was used to measure plasma glucose concentration. The FPG assay had a CV of 2.2%. In order to measure triglyceride (TG), a method utilising glycerol phosphate oxidase and enzymatic colorimetric techniques was employed. The coefficients of variation (CV) were determined to be 0.6% for inter‐assay and 1.6% for intra‐assay. The enzymatic colorimetric method evaluated total cholesterol (TC) by cholesterol esterase and cholesterol oxidase.

The high‐density lipoprotein cholesterol (HDL‐C) level was determined by using phosphotungstic acid to precipitate the lipoproteins containing apolipoprotein B. The coefficients of variation (CVs) for TC and HDL‐C were 0.5% and 2%, respectively. If the TG concentration was below 400 mg/dL, the Friedwald formula was applied to determine the LDL‐C level using the serum TC, TG, and HDL‐C concentrations [26].

#### 2.2.3 Carotid Artery Intima‐Media Thickness (cIMT) Assessment

During the study, the participants underwent an ultrasound using a Samsung Medison SonoAceR3 ultrasound machine with a 7.5e10‐MHz linear transducer. The intima‐media thickness of both the left and right common carotid arteries (CCA) was measured. Unaware of the participants' details, two radiologists examined them while lying on their backs with their necks extended and slightly turned to the opposite side of the examiner. Measurements were taken on both the left and right carotid, but ultimately, the LCCA was chosen based on earlier published articles in the literature [27, 28].

An initial carotid scan was conducted across the artery's course in the transverse plane to evaluate the participant's anatomy and locate any atherosclerotic plaques. The location of maximum wall thickening in the proximal or distal wall was also determined. Following this, a longitudinal scan was performed from various angles.

We measured arterial segments free of plaque and met specific criteria for optimal B‐mode imaging. These criteria included clear visualisation of the distal wall arterial interfaces with fully anechoic luminal content, depth optimization by scanning the arterial lumen in the centre of the image and setting the focal area at the level of the arterial lumen. This technique allowed us to obtain high‐quality grey‐scale images of the carotid artery, which we saved for later measurement. We adjusted the scanning depth to ensure that the arterial lumen was in the centre of the image and set the focal region accordingly.

During the cIMT procedure, a hypoechoic band was identified as the space between the echogenic intimal surfaces and other surfaces of the arterial wall. The distance between the front edge of the first and second echogenic lines of the peripheral walls of the terminal part of the common carotid artery was measured at three locations on both sides. The final measurement used the average of these values. Unfortunately, electrocardiography (ECG) was not performed during the scan, so the specific cardiac cycle during which the measurements were taken could not be determined.

cIMT of the carotid bulb and internal carotid artery was measured bilaterally sporadically in participants who met the optimal technique and imaging criteria. In the present study, we used the measurement of the peripheral wall of the left carotid artery (LCCA) to define the cIMT quartiles and analyse their relationship with the weight‐adjusted SMM quartiles.

To test the reliability agreement, two radiologists measured cIMT in 30 participants (66.7% female) with a mean age and BMI of 41.7 ± 10.7 years and 24.4 ± 5.5 kg/m^2^. The intraclass correlation coefficient (ICC) was used to evaluate the agreement of cIMT parameters between the two radiologists. The SPSS version 20 statistical package calculated ICC estimates and 95% confidence intervals based on a two‐way mixed effects model. The ICC result was reported as 0.79 with a 95% confidence interval of 0.55 to 0.90. ICC values range from 0 to 1, with values between 0.75 and 0.9 indicating good reliability [29]. Furthermore, there was a difference in cIMT between the readers, with a mean (SD) of 0.08 (0.12) mm.

#### 2.2.4 Body Composition Assessment

A portable bioelectrical impedance analyser (Model: InBody 570, InBody Co. Ltd. Seoul, Korea) was used to assess body composition. Participants were instructed to use electrolyte wipes to clean their palms and soles before standing for assessment. During the assessment, they stood with the foot electrodes in contact with their soles and held the paddle electrodes with their hands. Along with the recorded sex, height, weight and age, various segmental impedances were evaluated using eight‐electrode bioelectrical impedance analysis. This was done by measuring the impedances of different segments, such as right and left arms, trunk, and right and left legs, at frequencies of 5, 50 and 250 kHz from quadrupole electrodes. The participants took off their shoes and socks and put on light clothing. Then, they stood on the analyser platform and had their resistance to alternating current (500‐μA, 60/50 kHz) evaluated. The ‘standard’ option was used to interpret the data collected while standing still with arms at the sides. Using standard equations, BIA calculated the lean body mass (LBM) and percentage of body fat (PBF) [30]. One of the researchers conducted the evaluations. Also, SMMI was calculated with this relationship: skeletal muscle mass (kg)/height (m)^2^, and total body fat index from this relationship: total body fat mass (kg)/height (m)^2^ was calculated.

### 2.3 Statistical Analysis

All normally‐distributed continuous variables were expressed as mean ± standard deviation (SD). Otherwise, skewed continuous variables were presented by the median and interquartile range (IQR). Categorical baseline characteristics were reported as frequencies (percentages). Differences in participants' characteristics based on the SMM and SMMI quartile groups were assessed using the one‐way ANOVA test, Kruskal–Wallis's test, and Chi‐squared test for normally‐distributed quantitative, skewed quantitative and categorical variables, respectively. The association between SMM and SMMI quartile groups and high cIMT (above the 75th percentile) was explored by logistic regression analysis. Logistic regression analysis was conducted to calculate the odds ratio (OR) with corresponding 95% confidence intervals (CI). The logistic regression models were adjusted for potential confounders, including age, sex and other relevant covariates (including PBF, total cholesterol, Non‐HDL, HTN, physical activity, smoking status and family history of CVD). Additionally, linear regression analysis was performed to estimate the association between SMMI and SMM quartile groups and cIMT. The statistical analysis was conducted using the STATA software version 14 (STATA, College Station, TX, USA). A significance level of *p* < 0.05 (two‐tailed) was chosen for the study.

## 3 Result

The general characteristics of young adult men and women based on the skeletal muscle mass index (SMI) quartiles are presented in Table 1.

**TABLE 1:** General characteristics of participants.

Variables	Total (*n* = 795)	Men 434 (54.6%)					Women 361 (45.4%)				
		SMMI					SMMI				
		Q1 (*n*, %) 108 (24.9)	Q2 109 (25.1)	Q3 107 (24.7)	Q4 110 (25.3)	*p*	Q1 90 (24.9)	Q2 89 (24.7)	Q3 91 (25.2)	Q4 91 (25.2)	*p*
Age (years)	29.6 ± 3.9	29.3 ± 3.8	29.5 ± 4.0	29.8 ± 3.8	29.6 ± 3.7	0.821	28.9 ± 3.7	29.5 ± 4	30.1 ± 4.2	30.4 ± 3.8	0.045
Weight (kg)	75.6 ± 17.8	70.6 ± 12.5	81.1 ± 10.3	86.0 ± 10.2	98.9 ± 16.0	< 0.001	54.7 ± 7.8	60.4 ± 8.2	65.6 ± 8.5	80.2 ± 14.4	
Normal weight, *n* (%)	345 (43.4)	82 (75.9)	43 (39.4)	21 (19.4)	9 (8.2)	< 0.001	82 (91.1)	67 (76.1)	36 (40)	5 (5.5)	
Overweight, *n* (%)	309 (38.9)	23 (21.3)	60 (55)	65 (60.7)	41 (37.3)	< 0.001	8 (8.9)	18 (20.5)	46 (51.1)	48 (52.7)	
Obese, *n* (%)	139 (17.5)	3 (2.8)	6 (5.5)	21 (19.6)	60 (54.5)	< 0.001	0 (0.0)	3 (3.4)	8 (8.9)	38 (41.8)	
Body mass index (kg/m^2^)	26.2 ± 5.0	22.8 ± 3.4	25.8 ± 2.7	27.8 ± 3.1	31.2 ± 5.1	< 0.001	21.2 ± 2.6	23.3 ± 2.9	25.7 ± 3.0	30.8 ± 5.4	
Waist circumference (cm)	89.2 ± 12.4	84.6 ± 10.2	91.8 ± 9.0	95.0 ± 8.5	101.8 ± 11.8	< 0.001	76.5 ± 7.5	79.8 ± 7.8	85.2 ± 7.9	95.1 ± 11.5	
Abdominal obesity (%)	367 (46.2)	38 (35.2)	64 (58.7)	75 (70.1)	95 (86.4)	< 0.001	2 (2.2)	12 (13.5)	24 (26.4)	57 (62.6)	
SBP (mmHg)	107.3 ± 12.0	108.7 ± 9.7	111.7 ± 11.7	112.3 ± 9.9	114.6 ± 12.0	0.001	98.0 ± 8.3	99.8 ± 9.8	102.9 ± 10.9	106.6 ± 11.7	
DBP (mmHg)	73.2 ± 9.3	75.0 ± 7.8	75.6 ± 9.7	75.6 ± 9.5	76.9 ± 9.0	0.442	67.4 ± 7.6	69.7 ± 7.6	70.1 ± 8.7	73.0 ± 10.0	
Hypertension (%)	37 (4.7)	3 (2.8)	8 (7.3)	7 (6.5)	11 (10)	0.204	0 (0.0)	1 (1.1)	3 (3.3)	4 (4.4)	0.173
Diabetics (%)	16 (2.0)	2 (1.9)	0 (0)	0 (0)	3 (2.8)	0.144	1 (1.1)	4 (4.5)	3 (3.3)	3 (3.3)	0.612
Dyslipidemia (%)	364 (45.8)	32 (31.1)	35 (32.1)	45 (42.9)	62 (56.9)	< 0.001	38 (42.2)	38 (42.7)	49 (54.4)	65 (72.2)	
HDL‐C (mg/dL)	47.0 ± 10.8	47.4 ± 9.6	45.0 ± 8.5	43.8 ± 9.2	40.1 ± 10.4	< 0.001	53.6 ± 9.7	52.8 ± 11.8	49.8 ± 10.3	45.6 ± 9.8	
Non‐HDL (mg/dL)	127.1 ± 34.7	131.2 ± 34.9	132.8 ± 32.7	139.0 ± 38.2	137.8 ± 39.7	0.338	103.5 ± 25.1	115.9 ± 31.6	122.9 ± 30.2	125.5 ± 24.7	
Triglycerides (mg/dL)	102 (72–144.5)	105 (72–142)	111 (80–152)	129 (91–178)	147 (101–214)	< 0.001	72 (55–88)	83 (59–120)	89 (67–125)	103 (79–142)	
Total cholesterol (mg/dL)	173.8 ± 33.6	178.6 ± 33.9	177.8 ± 33.1	182.8 ± 36.6	177.9 ± 39.5	0.710	157.2 ± 27.7	168.6 ± 32.6	172.7 ± 30.4	171.2 ± 25.3	0.001
LDL (mg/dL)	102.8 ± 27.7	107.4 ± 27.9	107.5 ± 28.0	109.0 ± 30.8	105.1 ± 30.8	0.819	88.5 ± 22.4	97.5 ± 26.9	102.7 ± 24.9	101.6 ± 22.0	
FPG (mg/dL)	89.4 ± 11.3	92.0 ± 12.6	89.9 ± 8.7	90.7 ± 7.5	92.0 ± 17.8	0.512	85.7 ± 6.7	86.8 ± 13.1	88.0 ± 8.7	89.1 ± 7.9	0.090
Family history CVD (%)	18 (2.3)	5 (4.6)	0 (0)	1 (0.9)	6 (5.5)	0.032	1 (1.1)	1 (1.1)	2 (2.2)	2 (2.2)	0.886
Smoker, *n* (%)	173 (21.8)	28 (26.2)	38 (34.9)	33 (31.1)	45 (40.9)	0.129	7 (7.9)	10 (11.2)	7 (7.7)	5 (5.6)	0.585
Physical activity (METs min/week ≥ 600), (%)	251 (31.6)	43 (41)	34 (31.2)	34 (32.4)	26 (23.6)	0.059	42 (47.2)	29 (32.6)	23 (25.3)	20 (22)	0.001
Educational level > 12 years, *n* (%)	494 (62.1)	74 (68.5)	58 (53.2)	67 (62.6)	51 (46.4)	0.005	71 (78.9)	62 (69.7)	58 (63.7)	53 (58.2)	0.022
cIMT (mm)	0.553 ± 0.093	0.514 ± 0.096	0.533 ± 0.090	0.551 ± 0.102	0.550 ± 0.099	**0.015**	0.555 ± 0.080	0.567 ± 0.080	0.567 ± 0.076	0.596 ± 0.090	**0.008**
FM, kg (BFM)	24.9 ± 10.3	17.8 ± 8.7	23.0 ± 8.2	25.5 ± 8.3	31.1 ± 12.8	< 0.001	18.3 ± 5.1	21.9 ± 5.7	26.4 ± 6.3	35.7 ± 10.4	
FM (%) (PBF)	31.5 ± 8.8	23.9 ± 8.7	27.0 ± 6.5	28.1 ± 6.6	29.5 ± 8.2	< 0.001	32.6 ± 6.0	34.4 ± 5.7	37.8 ± 5.6	41.8 ± 6.2	
FFM, kg	53.0 ± 12.2	53.2 ± 5.3	60.2 ± 4.5	63.3 ± 4.5	71.1 ± 6.6	< 0.001	36.9 ± 3.2	40.8 ± 2.9	42.6 ± 3.4	48.4 ± 4.7	
FFM (%)	70.8 ± 9.6	76.8 ± 10.1	75.0 ± 7.8	74.4 ± 7.6	73.1 ± 9.7	0.018	68.4 ± 8.0	68.3 ± 7.9	65.7 ± 7.2	61.3 ± 7.2	
SMM, kg	29.6 ± 7.4	29.7 ± 3.1	34.0 ± 2.5	35.9 ± 2.6	40.6 ± 3.8	< 0.001	19.7 ± 1.9	22.1 ± 1.7	23.3 ± 2.0	23.0 ± 3.3	
Visceral fat level	11.1 ± 5.1	7.5 ± 4.6	9.9 ± 4.2	10.9 ± 4.2	13.4 ± 5.7	< 0.001	8.3 ± 3.2	10.0 ± 3.5	12.5 ± 4.0	16.5 ± 4.7	
Total body fat index, kg/m^2^	8.8 ± 3.8	5.7 ± 2.7	7.3 ± 2.5	8.2 ± 2.7	9.9 ± 4.2	< 0.001	7.1 ± 2.0	8.4 ± 2.3	10.3 ± 2.5	13.8 ± 4.1	
SMMI, kg/m^2^	10.1 ± 1.6	9.6 ± 0.7	10.8 ± 0.3	11.6 ± 0.2	12.7 ± 0.7	< 0.001	7.7 ± 0.5	8.5 ± 0.2	9.1 ± 0.2	10.3 ± 0.8	

*Note:* Continuous and categorical variables are represented as mean ± SD and frequency (percentage), respectively. Triglycerides are reported as median (IQR 25–75). Bold values indicate statistical significance (*p* < 0.05).

Abbreviations: CIMT, carotid intima‐media thickness; CVD, cardio vascular disease; DBP, diastolic blood pressure; FFM, fat‐free mass; FM, fat mass; FPG, fasting plasma glucose; HDL‐C, high‐density lipoprotein cholesterol; LDL‐C, low‐density lipoprotein cholesterol; SBP, systolic blood pressure; SSM, skeletal muscle mass; SSMI, skeletal muscle mass index.

In this cross‐sectional study, 795 participants, including 361 women (45.4%) and 434 men (54.6%), were investigated. In general, the mean age (SD), body mass index, cIMT, and SMMI were 29.6 years (3.9), 26.2 kg/m^2^ (5.0), 0.553 mm (0.093), and 10.1 kg/m^2^ (1.6), respectively. Other basic characteristics of the participants in the current study can be seen by sex in the SMMI quadrants in Table 1.

The prevalence of obesity and overweight, high blood pressure, and current smoking status was 17.5, 38.9, 4.7, 21.8% and 2.3%, respectively. The factors that had a negative relationship with SMMI quartiles in both sexes were HDL‐cholesterol and fat‐free mass percentage. In addition, the prevalence of people with normal weight had an inverse relationship with the quartiles of skeletal muscle mass, while this prevalence increased in overweight and obese people.

Table 1 shows the positive direct correlation and increase in cIMT with the quartiles of SMMI in both sexes, which significantly increased cIMT with the increase in SMMI. Regarding other body composition indices, including FM and FM percentage, FFM, SMM, visceral fat level and total body fat index, we also observed a direct increase in the relationship with the SMMI quartiles.

Table 2 shows the association between the quartiles of SMMI and high cIMT (above the 75th percentile). In general, the SMMI quartiles were directly associated with high cIMT in both sexes. In the unadjusted state, in both sexes, the risk of being exposed to high cIMT was significantly higher for the 4th quartile of SMMI than for the lowest quartile (in men, OR, 1.82; 95% CI, 1.03 to 3.22, and in women, OR, 2.00; 95% CI, 1.06 to 3.76). In men, the unadjusted state and model 1 (age‐adjusted model), this risk was significantly higher in the 3rd quartile too, while for women, this relationship was significant only in the 4th quartile. However, in model 1, this risk for the 4th quartile of SMMI reported after adjusting for age was for men and women (OR, 1.80; 95% CI, 1.02 to 3.20) and (OR, 2.17; 95% CI, 1.14 to 4.13), respectively.

**TABLE 2:** Association between SMMI and SMM with high CIMT according to sex.

Variables	Unadjusted	Model 1	Model 2
	OR (95% CI)	OR (95% CI)	OR (95% CI)
Men			
SMMI			
Q1	1	1	1
Q2	1.40 (0.78–2.50)	1.39 (0.78–2.49)	1.37 (0.74–2.54)
Q3	**1.98 (1.12–3.51)**	**1.95 (1.10–3.46)**	**1.86 (1.00–3.46)**
Q4	**1.82 (1.03–3.22)**	**1.80 (1.02–3.20)**	1.74 (0.92–3.30)
SMMI (continuous)	**1.24 (1.06–1.46)**	**1.24 (1.05–1.46)**	**1.23 (1.03–1.48)**
Women			
SMMI			
Q1	1	1	1
Q2	1.56 (0.82–2.96)	1.61 (0.84–3.08)	1.70 (0.85–3.37)
Q3	1.36 (0.71–2.60)	1.45 (0.76–2.80)	1.53 (0.74–3.19)
Q4	**2.00 (1.06–3.76)**	**2.17 (1.14–4.13)**	2.17 (0.97–4.84)
SMMI (continuous)	**1.25 (1.02–1.54)**	**1.30 (1.05–1.60)**	**1.33 (1.00–1.76)**
Men			
SMM			
Q1	1	1	1
Q2	1.37 (0.77–2.43)	1.34 (0.76–2.38)	1.36 (0.75–2.49)
Q3	1.65 (0.93–2.93)	1.63 (0.92–2.89)	1.66 (0.90–3.05)
Q4	**2.09 (1.19–3.67)**	**2.14 (1.22–3.77)**	**1.90 (1.02–3.52)**
SMM (continuous)	**1.06 (1.02–1.10)**	**1.06 (1.02–1.11)**	**1.06 (1.01–1.10)**
Women			
SMM			
Q1	1	1	1
Q2	1.29 (0.68–2.44)	1.35 (0.71–2.58)	1.42 (0.71–2.83)
Q3	1.37 (0.73–2.60)	1.45 (0.76–2.77)	1.69 (0.84–3.37)
Q4	**2.04 (1.09–3.82)**	**2.18 (1.16–4.12)**	**2.20 (1.05–4.58)**
SMM (continuous)	**1.09 (1.02–1.17)**	**1.10 (1.03–1.18)**	**1.11 (1.02–1.21)**

*Note:* Model 1, adjusted for age. Model 2, Model 1 plus further adjusted for family history CVD, hypertension, PBF, current smoking, non‐HDL, total cholesterol, physical activity. Bold values indicate statistical significance (*p* < 0.05).

When examining the continuous variables, a significant association was found between cIMT in the highest quartile and SMMI in both men (OR, 1.24; 95% CI, 1.06 to 1.46) and women (OR, 1.25; 95% CI, 1.02 to 1.54). This association was also significant after adjusting for age in Model 1 for both men (OR, 1.24; 95% CI, 1.05 to 1.46) and women (OR, 1.30; 95% CI, 1.05 to 1.60). It is noteworthy that after full adjustment for potential confounders (age, family history of CVD, hypertension, percent body fat, current smoking, non‐HDL and total cholesterol, and physical activity), the strength and statistical significance of the associations for the upper quartiles (Q3 and Q4) of SMMI were attenuated in both sexes. Specifically, in the fully adjusted model, only Q3 remained significantly associated with high cIMT in men, while the association for Q4 in women lost its statistical significance. However, this pattern was not consistent for SMMI as a continuous variable. The positive association between continuous SMMI and high cIMT remained significant in men and was marginally significant in women, indicating a more persistent linear relationship independent of the adjusted covariates (Table 2).

The association between skeletal muscle mass quartiles and high cIMT is reported in the second part of Table 2. As expected, the risk of exposure to high cIMT in the 4th quartile of SMM was significantly higher in both men and women (OR [95% CI] for men and women were reported as 2.09 [1.19–3.67] and 2.04 [1.09–3.82], respectively). Continuous variables were also reported to be significant in both sexes. After adjusting for age in Model 1, this significance was seen again for both sexes in the 4th quartile of SMM and its continuous variable. It is notable that in the fully adjusted model (Model 2), SMM as a continuous variable remained significantly associated with high cIMT in both women (OR, 1.11; 95% CI, 1.02–1.21) and men (OR, 1.06; 95% CI, 1.01–1.10). Additionally, the highest quartile (Q4) of SMM was significantly associated with the outcome in both sexes.

Table 3 describes the association of SMMI and SMM as quartiles and continuous variables with cIMT as a continuous variable in the linear and logistic multiple regression models. In both sexes, higher SMMI quartiles were positively associated with cIMT. This relationship was significant in men in both the 3rd and 4th quartiles in the unadjusted mode and Model 1.

**TABLE 3:** Association between SMMI and SMM with CIMT according to sex.

Variables	Unadjusted	Model 1	Model 2
	Beta (95% CI)	Beta (95% CI)	Beta (95% CI)
Men			
SMMI			
Q_1_	1	1	1
Q_2_	0.019 (−0.007, 0.044)	0.018 (−0.007, 0.044)	0.014 (−0.012, 0.040)
Q_3_	**0.037 (0.011, 0.063)**	**0.036 (0.010, 0.061)**	**0.030 (0.004, 0.057)**
Q_4_	**0.036 (0.010, 0.062)**	**0.035 (0.010, 0.060)**	0.027 (−0.001, 0.054)
SMMI (continuous)	**0.012 (0.005, 0.019)**	**0.012 (0.005, 0.019)**	**0.010 (0.002, 0.018)**
Women			
SMMI			
Q_1_	1	1	1
Q_2_	0.013 (−0.011, 0.036)	0.012 (−0.012, 0.036)	0.014 (−0.010, 0.037)
Q_3_	0.012 (−0.011, 0.036)	0.012 (−0.012, 0.035)	0.010 (−0.015, 0.035)
Q_4_	**0.041 (0.017, 0.064)**	**0.040 (0.016, 0.064)**	**0.030 (0.002, 0.058)**
SMMI (continuous)	**0.015 (0.007, 0.023)**	**0.015 (0.007, 0.023)**	**0.013 (0.003, 0.023)**
Men			
SMM			
Q_1_	1	1	1
Q_2_	0.015 (−0.010, 0.040)	0.013 (−0.012, 0.038)	0.012 (−0.013, 0.038)
Q_3_	0.025 (−0.001, 0.050)	0.023 (−0.002, 0.049)	0.020 (−0.006, 0.047)
Q_4_	**0.036 (0.011, 0.061)**	**0.038 (0.013, 0.063)**	**0.030 (0.003, 0.056)**
SMM (continuous)	**0.003 (0.001, 0.005)**	**0.003 (0.001, 0.005)**	**0.003 (0.001, 0.005)**
Women			
SMM			
Q_1_	1	1	1
Q_2_	0.017 (−0.007, 0.040)	0.016 (−0.008, 0.039)	0.014 (−0.009, 0.038)
Q_3_	0.019 (−0.005, 0.043)	0.018 (−0.005, 0.042)	0.020 (−0.004, 0.043)
Q_4_	**0.043 (0.019, 0.063)**	**0.042 (0.018, 0.066)**	**0.034 (0.008, 0.038)**
SMM (continuous)	**0.005 (0.002, 0.007)**	**0.004 (0.002, 0.007)**	**0.004 (0.001, 0.007)**

*Note:* Model 1, adjusted for age. Model 2, Model 1 plus further adjusted for family history CVD, hypertension, PBF, current smoking, non‐HDL, total cholesterol, physical activity. Bold values indicate statistical significance (*p* < 0.05).

For the continuous variable of SMMI in both sexes, beta (95% CI) corresponding to unadjusted was 0.012 (0.005, 0.019) in men and 0.015 (0.007, 0.023) in women (Table 3). This significance was still observed in both sexes after adjusting for age. It should be noted that after full adjustment for confounders in Model 2, the strength of this association was slightly attenuated, particularly in men, yet it remained statistically significant in both sexes (Table 3).

## 4 Discussion

The results of our study provide valuable insights into the association between SMMI and cardiovascular health indicators, specifically cIMT and high cIMT, in the young adult population. This age group is of particular interest as it represents a critical period for the establishment of lifestyle habits and the development of early markers of CVD. Our findings demonstrate that even in young adults, higher levels of SMM, as indicated by SMMI quartiles, are associated with increased cIMT and high cIMT. These results highlight the importance of considering SMM as a potential component for early cardiovascular changes, beyond traditional risk factors, in this specific age group.

Some studies have specifically investigated the relationship between body composition variables, particularly body composition, and cardiovascular outcomes. In a comprehensive prospective cohort study involving 356,590 participants from the UK Biobank, aged 40 to 69 years, it was observed that in men, there existed a positive association between appendicular skeletal muscle mass (SMM) and the incidence of cardiovascular disease (CVD) (hazard ratio [HR] per 1 standard deviation [SD] 1.07; 95% CI, 1.06–1.09). Notably, a curvilinear association was identified in women. More robust positive associations with cardiovascular disease (CVD) were evident for fat mass (FM), with hazard ratios (HRs) per standard deviation (SD) of 1.20 (95% CI, 1.19–1.22) in men and 1.25 (95% CI, 1.23–1.27) in women. Interestingly, even within tertiles of fat mass, the associations between appendicular skeletal muscle mass (aSMM) and the risk of CVD remained largely intact [1]. Some studies have also examined the correlation between the cIMT, as a cardiovascular surrogate marker, and indicators of body composition. For instance, Agbaje et al., in a cohort study revealed that alterations in cIMT during the transition from adolescence to young adulthood could be attributed to structural adaptations in the arteries caused by elevated lean mass and systolic blood pressure (BP) in childhood. These associations remained significant even after accounting for other factors such as cardiometabolic risks, lifestyle factors, and vascular organ damage. Moreover, the study revealed a cross‐sectional association between higher lean mass and increased cIMT at 24 years of age. The study also discovered that fat mass and BMI exhibited an inverse relationship with cIMT at the same age, after accounting for lean mass. Importantly, the research demonstrated that the cumulative exposure to high lean mass between the ages of 9 and 24 years independently and significantly predicted increased cIMT at age 24 years old [13]. Our findings are consistent with the studies, which highlighted a robust correlation between higher BMI, an indicator of adiposity, and elevated arterial stiffness [31] as well as cIMT [32, 33, 34]. The research conducted by Oren and colleagues [32] on a cohort of 750 healthy young individuals, aged 27–30 years, revealed that a one standard deviation (SD) rise in BMI during adolescence correlated with a 2.3‐μm increase in mean common cIMT among young adults. This association remained significant even after adjusting for factors such as gender, age during adolescence, blood pressure during adolescence, puberty stage, and lumen diameter. Controlling for adult BMI diminished the connection (linear regression coefficient = 0.9 μm per standard deviation; 95% CI: −0.3; 2.2), given that a significant proportion of overweight and obese adolescents maintained their overweight status or progressed to obesity in early adulthood. Individuals who consistently stayed within the higher BMI range from adolescence to young adulthood exhibited a significantly greater common cIMT compared to those who experienced relative weight loss over time (mean difference 14.7 μm; *p* < 0.001). These latter showed similar cIMT values as individuals with constant low BMI. Our findings are consistent with the above‐mentioned studies that were conducted on young adult age groups, showing a positive association between skeletal muscle or lean body mass and cIMT.

It is interesting to note that our findings differ from some previous studies that suggest a collaboration between increased skeletal muscle mass and better cardiovascular health outcomes. A study examining a large group of middle‐aged Koreans discovered that those with low relative muscle mass had a higher incidence of subclinical coronary artery disease, along with an increasing degree of coronary artery calcification proportional to their muscle mass [35]. Additionally, a Korean population‐based study revealed a significant correlation between low relative muscle mass, measured by the skeletal muscle index (SMMI), and an elevated risk of cardiovascular disease [5, 12]. Also, a cross‐sectional study in Japan revealed an association between low relative muscle mass and increased arterial stiffness in women, suggesting a potential common pathway between low muscle mass and atherosclerosis [5]. Similarly, a study conducted in Korea by Heo et al. observed that reduced skeletal muscle mass was independently linked to the highest quartile of cIMT and the presence of carotid artery plaque among middle‐aged Korean men who had lower body mass indexes (BMIs). At the same time, regardless of BMI, no significant association was observed in women [22]. It is worth noting that the average age of participants in the studies, as mentioned earlier, typically exceeded 40 years, which differs from our study and similar investigations that focused on young and middle‐aged individuals. This divergence may reflect the initiation of the atherosclerosis process as opposed to the arterial wall remodelling observed in younger populations. Additionally, as we have discussed in a large Biobank study [1] with a long follow‐up period, assessed cardiovascular outcome directly, not surrogate biomarkers, body composition over time causes changes in the cIMT.

One plausible physiological mechanism that may link higher appendicular skeletal muscle mass (ASMM) with an increased risk of cardiovascular disease (CVD) is a higher circulating blood volume. This can lead to increased cardiac output, elevated systolic blood pressure, and a higher risk of heart failure, particularly observed in individuals with obesity [36, 37, 38]. Interestingly, a recent literature review has shed light on the role of lean mass in metabolic health, suggesting the possibility of publication bias, especially when unexpected results are obtained [39]. Longitudinal studies are required to gain a deeper understanding of the relationship between skeletal muscle mass and cardiovascular health outcomes. These studies can explore the impact of skeletal muscle mass on CVD risk in young adults over an extended period and investigate the potential benefits of interventions, such as resistance training, to enhance skeletal muscle mass and mitigate cardiovascular risk. Furthermore, recent evidence suggests that skeletal muscle may possess endocrine functions and release bioactive molecules known as myokines, which could affect cardiovascular health [40, 41, 42]. Investigating the intricate interplay between skeletal muscle, myokines, and cardiovascular health is an important area for further research.

The positive association observed between SMMI/SMM and cIMT, independent of major confounders including percent body fat, invites careful interpretation. While skeletal muscle is traditionally viewed as metabolically beneficial, our findings in this young adult cohort suggest that higher muscle mass may not be independently cardioprotective in the context of early‐stage atherosclerosis. A plausible explanation aligning with our data is the ‘obesity paradox’ phenomenon in body composition, where high absolute muscle mass often coexists with high adiposity, especially in younger, non‐sarcopenic populations. In such a phenotype, the observed vascular thickening (increased cIMT) may be primarily driven by the adverse metabolic and hemodynamic effects of concurrent excess fat mass, with muscle mass acting as a bystander or even a compensatory response. This is supported by the positive correlations seen in our data between SMMI quartiles and other adiposity indices (Table 1). Therefore, our results underscore that high muscle mass should not be equated with optimal cardiovascular health when evaluated in isolation from adiposity.

Our study possesses several key strengths that enhance its scientific value. It employs a rigorous cross‐sectional design with an optimal sample size that increases the reliability and generalizability of our findings. Our study is the first conducted in the region among the young adults. It is important to acknowledge the limitations of our study. While we tried to adjust for various lifestyle confounders, it is important to acknowledge the possibility of residual confounding concerning nutrition and other lifestyle factors in our study. We did not examine inflammatory factors, which could potentially influence the association between skeletal muscle mass and cIMT. Our study design was cross‐sectional, limiting our ability to establish causality or determine the temporal sequence of events. Lastly, we utilised BIA for estimating body composition. While BIA is a commonly used and non‐invasive approach, DXA provides a more precise assessment of body composition. However, a correlation has been reported between the body composition data of BIA and the data of DXA, and it was suggested that BIA and DXA methods are interchangeable at a population level [43].

## 5 Conclusion

Overall, this study demonstrates a significant association between higher skeletal muscle mass and increased cIMT in young adults, a relationship that persisted after adjustment for key confounders including percent body fat. Contrary to the conventional view of muscle mass as an unequivocally protective factor, our findings suggest that in this specific young population, higher muscle mass may serve as a marker for, or coexist with, a body composition phenotype (high muscle‐high fat) that is associated with early vascular changes. This indicates that the cardiovascular implications of muscle mass cannot be assessed in isolation and are likely dependent on the concomitant level of adiposity. Therefore, public health messages and clinical interventions should focus on promoting a healthy overall body composition profile rather than increasing muscle mass per se. Future longitudinal studies are essential to determine the causality and directionality of this relationship, and to elucidate whether interventions that reduce adiposity while preserving or optimising muscle mass can mitigate the risk of progressive atherosclerosis.

## Author Contributions

M.N., B.A., M.V. and F.H. designed and wrote the manuscript. M.M., and M.N. performed interpretation and critical revision of the manuscript. F.H., M.V., P.D., F.A., and B.A. critically revised the manuscript. All authors read and approved the final version.

## Funding

The authors have nothing to report.

## Ethics Statement

According to the 2013 Helsinki Declaration guidelines, the protocol of this study was approved by the Medical Ethics Committee of Shahid Beheshti University of Medical Sciences (approval number: IR.SBMU.ENDOCRINE.REC.1401.079). All participants signed an informed consent form.

## Consent

All participants signed an informed consent form.

## Conflicts of Interest

The authors declare no conflicts of interest.

## Data Availability

The data that support the findings of this study are available on request from the corresponding author, F.H.
